# PI3K and PKC contribute to membrane depolarization mediated by α_2_-adrenoceptors in the canine isolated mesenteric vein

**DOI:** 10.1186/1472-6793-5-9

**Published:** 2005-06-15

**Authors:** Ilia A Yamboliev, Violeta N Mutafova-Yambolieva

**Affiliations:** 1 Department of Pharmacology and Center of Biomedical Research Excellence, University of Nevada School of Medicine, Reno, Nevada 89557, USA; 2 Department of Physiology and Cell Biology, University of Nevada School of Medicine, Reno, Nevada 89557, USA

## Abstract

**Background:**

Norepinephrine (NE), a classic neurotransmitter in the sympathetic nervous system, induces vasoconstriction of canine isolated mesenteric vein that is accompanied by a sustained membrane depolarization. The mechanisms underlying the NE-elicited membrane depolarization remain undefined. In the present study we hypothesized that phosphatidylinositol 3-kinase (PI3K) and protein kinase C (PKC) are involved in the electrical field stimulation (EFS)-induced slow membrane depolarization (SMD) in canine isolated mesenteric vein. EFS (0.1–2 Hz, 0.1 ms, 15V, 10 s)-induced changes in the membrane potential were recorded with a conventional intracellular microelectrode technique and evaluated in the absence and presence of inhibitors of neuronal activity, α-adrenoceptors, membrane ion channels, PI3K, inositol 1,4,5-triphosphate (InsP3) receptors, and PKC. Activation of PI3Kγ and PKCζ in response to exogenous NE and clonidine in the absence and presence of receptor and kinase inhibitors were also determined.

**Results:**

Contractile responses to NE and clonidine (0.05 – 10 μM) were significantly diminished in the presence of yohimbine (0.1 μM). Exogenous NE (0.1 μM) and clonidine (1 μM) elicited SMD. The resting membrane potential of canine mesenteric vein smooth muscle cells was -68.8 ± 0.8 mV. EFS elicited a biphasic depolarization comprised of excitatory junction potentials and SMD that are purinergic and adrenergic in nature, respectively. The magnitude of the SMD in response to EFS at 0.5 Hz was 9.4 ± 0.7 mV. This response was reduced by 65–98% by the fast Na^+ ^channel inhibitor tetrodotoxin (1 μM), by the inhibitor of N-type Ca^2+ ^channels ω-conotoxin GVIA (5 nM), the non-selective α-adrenoceptor blocker phentolamine (1 μM), the selective α_2_-adrenoceptor blocker yohimbine (0.1 μM), the ion channel inhibitors niflumic acid (NFA, 100 μM), 5-nitro-2-(3-phenylpropylamino) benzoic acid (NPPB, 30 μM), 4,4'-diisothiocyanatostilbene-2,2'-disulfonic acid (DIDS, 200 μM), and Gd^3+ ^(30 μM), and the PI3K inhibitors wortmannin (100 nM) and LY-294002 (10 μM). The SMD remained unchanged in the presence of the L-type Ca^2+ ^channel blocker nicardipine (1 μM) and the InsP_3 _receptor blockers 2-aminoethoxydiphenylborate (2APB, 50 μM) and xestospongin C (3 μM). The inhibitor of PKC chelerythrine (1 μM), but not calphostin C (10 μM), diminished the SMD. Exogenous NE and clonidine (1 μM each) activated both PI3Kγ and PKCζ, and the activation of these kinases was abolished by preincubation of tissue with the α_2_-adrenoceptor blocker yohimbine.

**Conclusion:**

Neuronally-released NE stimulates smooth muscle α_2_-adrenoceptors and activates PI3K and atypical PKC in the canine mesenteric vein. Events downstream of PKC lead to SMD and vasoconstriction. This represents a novel pathway for NE-induced membrane depolarization in a vascular smooth muscle preparation.

## Background

Norepinephrine (NE), a classic neurotransmitter in the sympathetic nervous system, is released from adrenergic varicosities of stimulated postganglionic nerve terminals, activates postjunctional α-adrenoceptors and gives rise to a slow membrane depolarization (SMD) and contraction [1,29]. The NE-induced SMD represents an important mechanism of excitation-contraction coupling in blood vessels however the signaling pathways underlying the NE-elicited SMD in vascular smooth muscle remain undefined.

One well-documented pathway downstream of activated G-protein coupled receptors (GPCRs) includes dissociation of G_αβγ _trimers and production of G_α _monomer and G_βγ _dimer, and involvement of the latter proteins in signal transduction events downstream of α-adrenoceptors. For example, G_α _mediates activation of phospholipase C (PLC), hydrolysis of phosphatidylinositol 4,5-bisphosphate (PI4,5P_2_), and generation of second messengers including inositol 1,4,5-triphosphate (InsP_3_) and diacylglycerol, DAG [20]. These second messengers then mediate signal transduction events leading to activation of ion channels. InsP_3 _has the capacity to release cytosolic Ca^2+ ^from intracellular stores, which then activates Ca^2+^-activated Cl^- ^channels (ClC_Ca_) and membrane depolarization, required for opening of voltage-operated calcium channels (VOCC) and Ca^2+ ^influx. DAG, on the other hand, activates non-selective cation channels (NSCC) in rabbit portal vein [17]. In addition, it becomes increasingly clear that G_βγ_ dimers can initiate intracellular signal transduction events as well. Phosphatidylinositol 3-kinase-γ (PI3Kγ), a member of class IB PI3Ks, was identified as a major effector of G_βγ _in various cell and tissue preparations [13,18]. Lipid products of the PI3Ks, phosphatidylinositol 3,4-bisphosphate (PI3,4P_2_) and phosphatidylinositol 3,4,5-trisphosphate (PI3,4,5P_3_), function as second messengers and can directly affect the activity of the membrane ion channels CFTR [12] and voltage-gated potassium channels [19]. Alternatively, PI3,4P_2 _and PI3,4,5P_3 _can modulate membrane ion channels via activation of PKC isozymes [6,25]. For example, G_βγ_, PI3Kγ, and atypical PKC were shown to link activation of G-protein coupled M_2_-muscarinic receptors to metabotropic Ca^2+ ^and voltage-independent Cl^- ^channels in *Xenopus *oocytes [31]. It was also demonstrated that PI3K mediates activation of L-type Ca^2+ ^channels upon stimulation of M_2_-muscarinic receptors in rabbit portal vein myocytes [3] and α_2_-adrenoceptor induced vasoconstriction in porcine palmar lateral vein [27]. These studies imply that activation of GPCRs could activate membrane ion channels and SMD via PI3K-dependent mechanisms. To our knowledge, however, coupling of α-adrenoceptors to PI3Kγ and membrane depolarization in vascular smooth muscles has not yet been reported.

We used canine isolated mesenteric vein to test the hypothesis that EFS-induced SMD is mediated by PI3K and PKC. Our results demonstrate both nerve stimulation and exogenous NE-mediated activation of α_2_-adrenoceptors, PI3K and PKC, and suggest a role for these kinases for the activation of membrane ion channels (e.g., ClC_Ca _and/or NSCC) and development of SMD.

## Results

### α_2_-Adrenoceptors mediate vasoconstriction and membrane depolarization in canine isolated mesenteric vein

**Figure 1:**
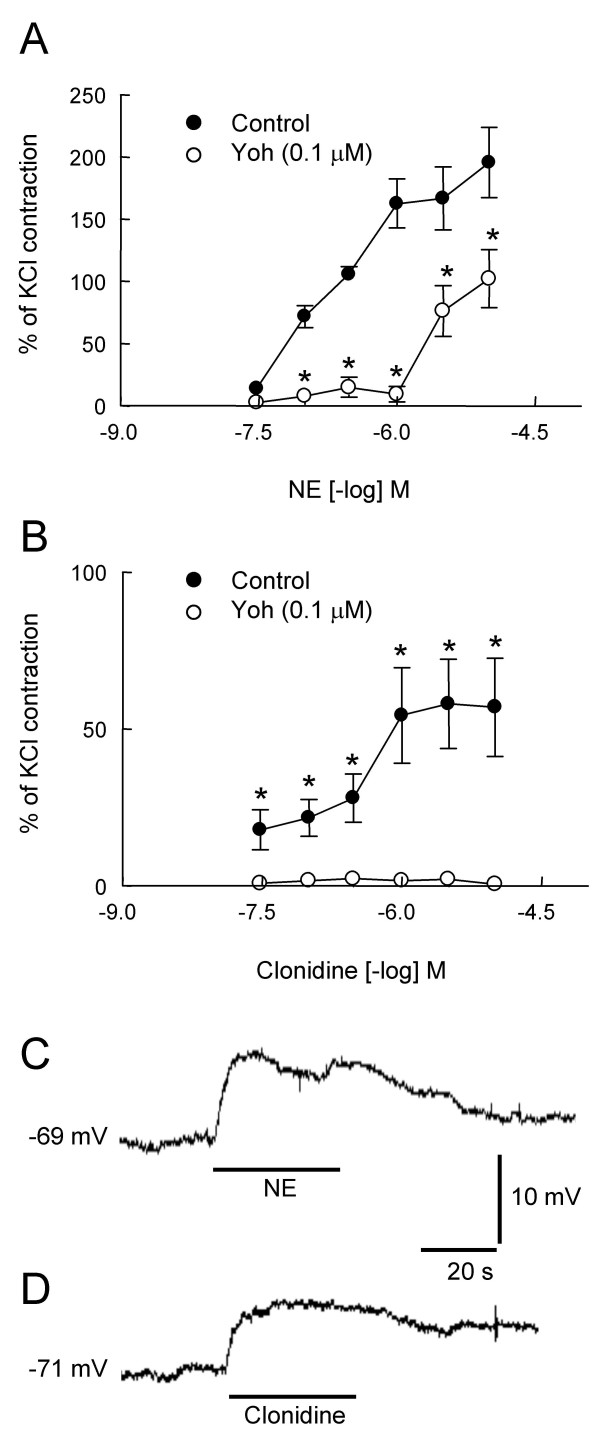
**The α_2_-adrenoceptor blocker yohimbine reduces the contractile responses of canine isolated mesenteric vein to norepinephrine (NE) and clonidine**. Contractile responses of control and yohimbine-incubated tissues (0.1 μM, 30 min) to exogenous NE (A) and clonidine (0.05–10 μM each). Contractile responses were expressed as percentage of the contractile response to 70 mM KCl. Data are presented as mean ± SEM, n = 4–8. *, P < 0.05. Representative traces of intracellular recordings of membrane potential changes elicited by NE (0.1 μM, 30 s, panel C) or clonidine (1 μM, 30 s, panel D). Scale bars apply to traces C and D.

Cumulative application of exogenous NE and clonidine (0.05 μM-10 μM) resulted in concentration dependent contractile responses (Fig. 1A and 1B). In the presence of the selective α_2_-adrenoceptor antagonist yohimbine (0.1 μM) the contractile responses to 0.05–1 μM NE were virtually abolished, whereas the responses to 5 and 10 μM NE were significantly reduced (Fig. 1A). Yohimbine abolished the mechanical responses to all clonidine concentrations (Fig. 1B). These results indicate that α_2_-adrenoceptors are particularly important for NE-mediated vasoconstriction in this blood vessel. Moreover, exogenous application of either NE (0.1 μM, Fig. 1C) or clonidine (1 μM, Fig. 1D) elicited SMD, suggesting that α_2_-adrenoreceptors may be involved in the membrane potential changes in response to NE that is released upon EFS.

### EFS gives rise to a biphasic membrane depolarization

**Figure 2:**
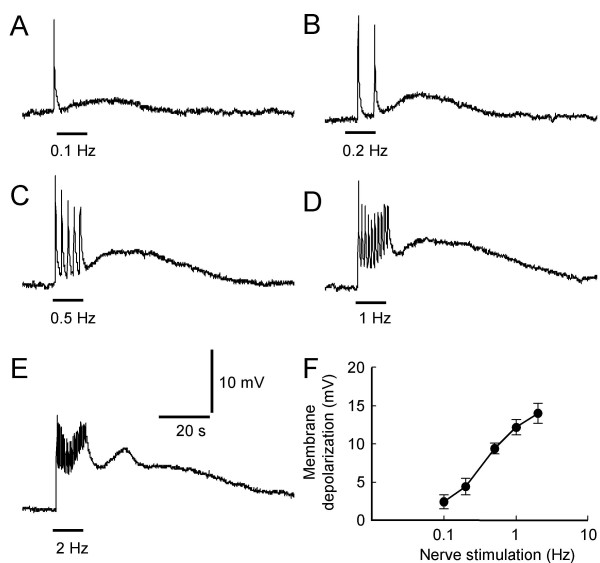
**Typical biphasic membrane depolarization of canine isolated mesenteric vein**. Representative intracellular recordings depicting EFS-induced (0.1 ms, 13V, 10 s, 0.1–2 Hz) biphasic membrane depolarizations, consisting of excitatory junction potentials and slow membrane depolarization (A-E). The amplitude of the slow membrane depolarization increases with the increase of stimulation frequencies (F). Mean ± SEM, n = 4–12. Scale bars apply to traces A-E.

**Figure 3:**
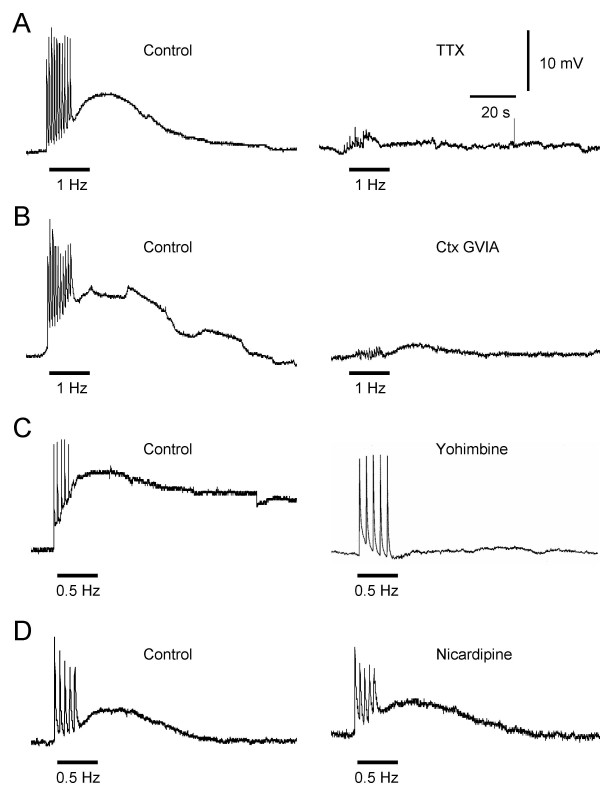
**Tetrodotoxin (TTX), ω-conotoxin GVIA (Ctx GVIA) and yohimbine, but not nicardipine, inhibit the EFS-induces slow membrane depolarization of canine isolated mesenteric vein**. Intracellular recordings of membrane potential in response to EFS (0.1 ms, 13 V, 10 s) in canine isolated mesenteric veins preincubated for 30 min prior to and throughout the experiment with 1 μM TTX (A), 5 nM ω-conotoxin GVIA (Ctx GVIA, B), 0.1 μM yohimbine (C) and 1 μM nicardipine (D). Scale bars apply to all traces.

The resting membrane potential of isolated canine mesenteric vein segments averaged -68.8 ± 0.8 mV (n = 78). EFS (0.1–2 Hz, 0.1 ms, 15 V, 10 s) gave rise to a characteristic biphasic depolarization of the cell membrane (Fig. 2A–E), composed of a fast excitation junction potential (EJP) of presumed purinergic nature, and a SMD of adrenergic origin. The amplitude of the SMD increased with the frequency of EFS (Fig. 2A–F). To better understand the processes that govern membrane depolarization, we exposed blood vessels to the fast Na^+ ^channel blocker tetrodotoxin (TTX, 1 μM) or to the neuronal N-type Ca^2+ ^channel blocker ω-conotoxin GVIA (ω-CtX GVIA, 5 nM). Both drugs virtually abolished the electrical responses to EFS (Fig. 3A, B), suggesting that the EFS-elicited SMD requires activation of postganglionic nerve terminals and release of a neurotransmitter substance. Phentolamine (1 μM) significantly reduced the SMD in response to 0.5 Hz EFS to 2.1 ± 0.5 mV (n = 3, P < 0.05, not shown), consistent with the possibility that NE-mediated activation of α-adrenoceptors is the primary cause for the SMD in blood vessels [14,29]. Furthermore, the selective α_2_-adrenoceptor antagonist yohimbine (0.1 μM, Fig. 3C), but not the selective α_1_-adrenoceptor antagonist prazosin (1 μM; 8.9 ± 0.9, n = 3, not shown), significantly reduced the SMD in response to 0.5 Hz EFS from 9.4 ± 0.7 (n = 14) to 0.8 ± 0.5 mV (n = 3, P < 0.05), indicating that the EFS-evoked SMD is mediated primarily by α_2_-adrenoceptors. Nicardipine (1 μM) abolished the contraction elicited by 70 mM KCl (not shown), indicating that L-type Ca^2+ ^channels are present in this blood vessel. However, the EFS-induced SMD remained unchanged in tissues preincubated with nicardipine (Fig. 3D), suggesting that opening of L-type Ca^2+ ^channels is not required for the membrane depolarization.

### The EFS-induced slow membrane depolarization is sensitive to NFA, NPPB, DIDS and Gd^3+^

**Figure 4:**
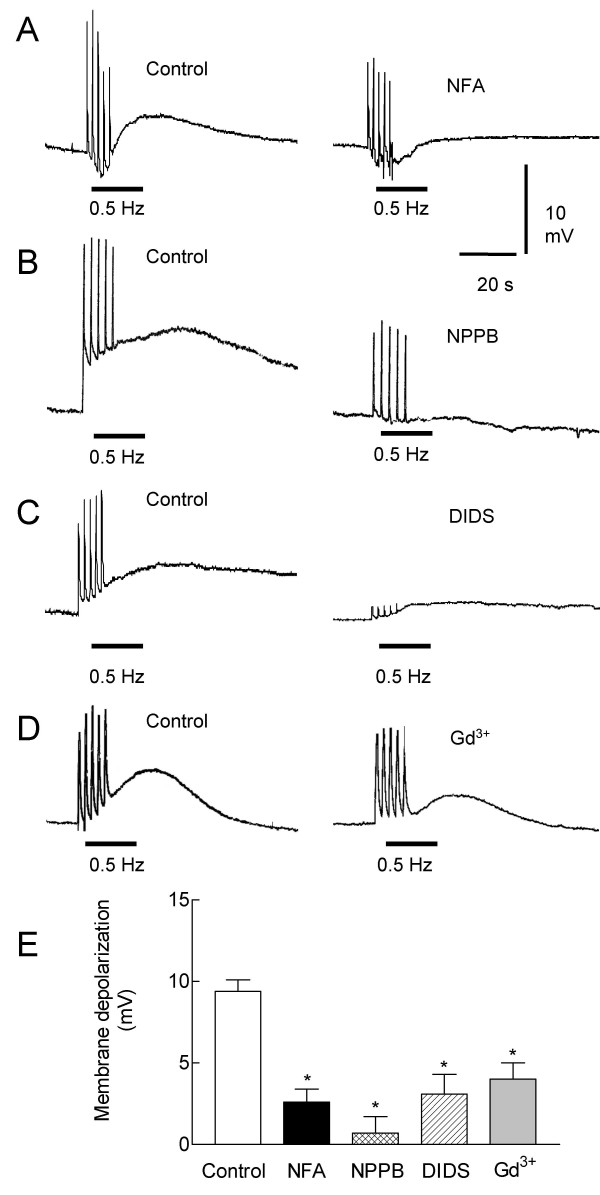
**The EFS-induced slow membrane depolarization of canine isolated mesenteric vein is reduced by various ion channel inhibitors**. Intracellular recordings of membrane potential in response to EFS (0.1 ms, 13 V, 10 s, 0.5 Hz) in canine isolated mesenteric veins preincubated for 60 min prior to and throughout the experiment with 100 μM NFA (A), 30 μM NPPB (B), 200 μM DIDS (C) and 30 μM Gd^3+ ^(D). The experimental data are presented as mean ± SEM, n = 3–4 (E). *, *P *< 0.05 *versus *control. Scale bars apply to traces A-D.

To test possible contribution of ion channels in the EFS-induced SMD, we incubated veins with the commonly used non-selective cation channel (NSCC) and Cl^- ^channel inhibitors NFA (100 μM), NPPB (30 μM), and DIDS (200 μM) [16,26]. These inhibitors reduced the 0.5 Hz EFS-elicited SMD by 73% (Fig. 4A and 4E), 92% (Fig. 4B and 4E) and 67% (Fig. 4C and 4E, n = 3–14), respectively. DIDS also reduced the excitation junction potentials (Fig. 4C), which may be due to blockade of P2X receptors, as suggested previously [2]. The NSCC inhibitor Gd^3+ ^reduced the SMD by 60% (Fig. 4D and 4E, n = 3). Together, our results indicate that the SMD in canine mesenteric vein depends on activation of membrane ion channels (e.g. ClC_Ca _and/or NSCC).

### InsP_3_ receptors are not required for the EFS-elicited slow depolarization

**Figure 5:**
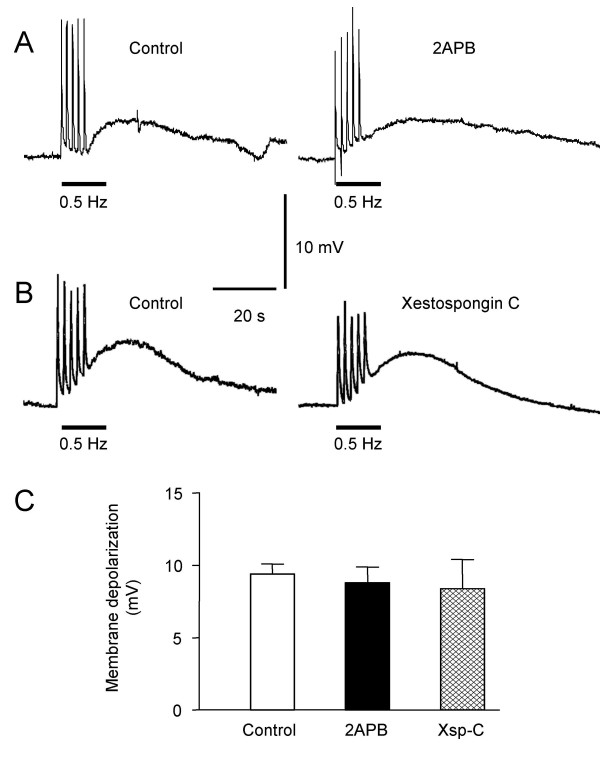
**2APB and xestospongin C have no effect on the EFS-induced slow membrane depolarization of canine isolated mesenteric vein**. Intracellular recordings of membrane potential in response to EFS (0.1 ms, 13 V, 10 s, 0.5 Hz) in canine isolated mesenteric veins preincubated for 60 min prior to and throughout the experiment with 50 μM 2APB (A) or 30 μM xestospongin C (B). The experimental data are presented as mean ± SEM, n = 3–4 (C). Scale bars apply to traces A and B.

To determine whether EFS-induced SMD is mediated by InsP_3 _receptors, veins were incubated with 2APB (50 μM), a selective inhibitor of InsP_3 _receptors and hence of GPCR-PLC-InsP_3_-mediated Ca^2+ ^release from sarcoplasmic reticulum. Surprisingly, SMD to 0.5 Hz EFS remained unaffected by 2APB (Fig. 5A and 5C). To test the possibility that the lack of effect of 2APB was due to poor membrane permeability, we carried out mechanical experiments with mesenteric veins, incubated with 2APB (50 μM) prior to incubation with increasing concentrations of the α_1_-adrenoceptor agonist methoxamine (0.1–100 μM). 2APB produced a significant rightward shift of the concentration-response curve of methoxamine (not shown) and a decrease of the EC50 from 6.2 ± 0.3 μM (n = 10) to 5.1 ± 0.2 μM (n = 6). These results suggest that activation of InsP_3_ receptors may be unnecessary for the SMD. This possibility was also confirmed by the inability of another inhibitor of InsP_3_ receptors, xestospongin C (3 μM), to reduce the EFS-evoked SMD (Fig. 5B and 5C, n = 3). Therefore, the EFS-mediated SMD may not require release of Ca^2+ ^from InsP_3 _receptor-operated stores.

### PI3K blockers reduce the EFS-elicited slow depolarization

**Figure 6:**
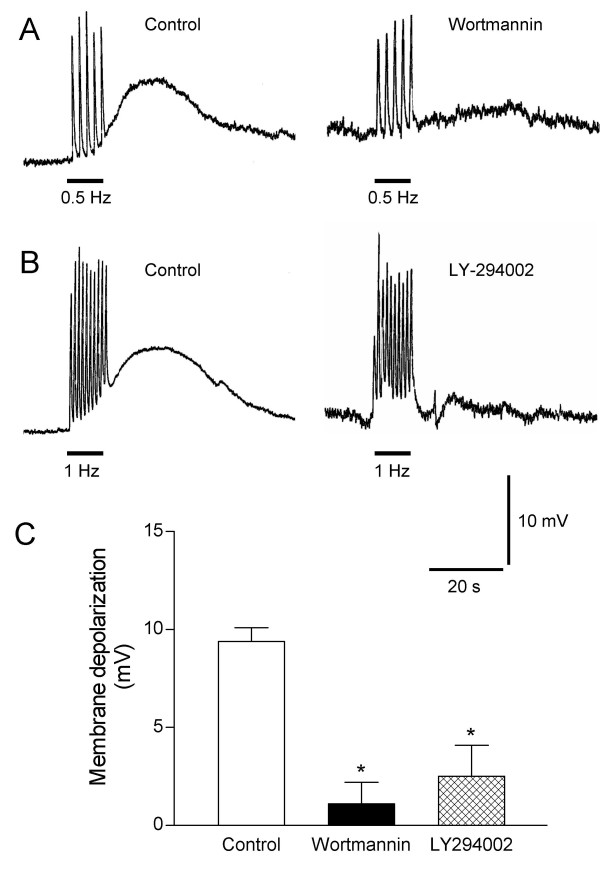
**The PI3K inhibitors wortmannin and LY-294002 inhibit the EFS-induced slow membrane depolarization in canine isolated mesenteric vein**. Intracellular recordings of membrane potential in response to EFS (0.1 ms, 13 V, 10 s) in canine isolated mesenteric veins preincubated for 60 min prior to and throughout the EFS with 100 nM wortmannin (A) or 10 μM LY-294002 (B). The experimental data are presented as mean ± SEM, n = 3–4 (C). *, *P *< 0.05 vs control. Scale bars apply to traces A and B.

To test whether PI3Ks are involved in the EFS-induced SMD, we incubated tissue strips with the PI3K inhibitors wortmannin (100 nM) and LY-294002 (10 μM). Both inhibitors significantly reduced the SMD elicited by 0.5 Hz EFS from 9.4 ± 0.7 mV in controls to 1.0 ± 1.1 and 2.5 ± 1.6 mV, respectively (Fig. 6), indicating that activation of PI3Ks is required for the EFS-induced activation of ion channels and SMD. Incubation of veins with exogenous NE (100 nM) induced 14.1 ± 3.0 mV SMD, which was reduced to 3.0 ± 2.0 mV (n = 3) in the presence of wortmannin (100 nM, not shown), suggesting that both EFS and exogenous NE-induced SMD are mediated by PI3K.

### Exogenous NE and clonidine activate PI3Ks

**Figure 7:**
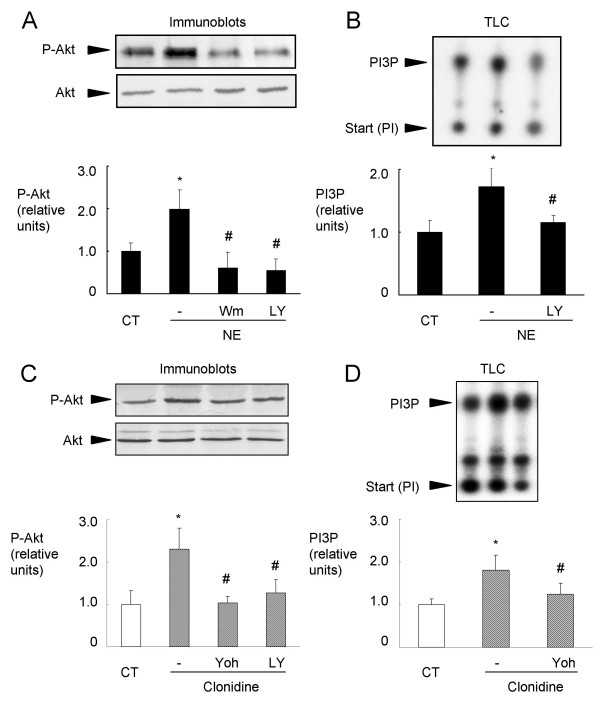
**Exogenous NE and clonidine activate PI3Ks in canine isolated mesenteric vein**. Immunoblots of Akt and phospho-Akt (P-Akt) in untreated control tissues (CT), and in tissues treated with NE (1 μM, 3 min) alone or after 60-min preincubation with 100 nM wortmannin, Wm, or 10 μM LY-294002, LY (A). Immune blots of P-Akt in veins incubated with clonidine (1 μM, 3 min) in the absence or after 60-min preincubation with 0.1 μM yohimbine, Yoh (C). The phospho-Akt (P-Akt) band densities were normalized to the respective Akt band densities, and the P-Akt/Akt ratios of treated tissues were presented relative to the ratios of non-treated tissue controls (bar graphs in A and C, n = 3). Thin-layer chromatography (TLC) separation of PI3P, obtained upon *in vitro *phosphorylation of PI by immunoprecipitated PI3Kγ. PI3Kγ activation was assessed in untreated (control) tissue (CT) and in tissues incubated for 3 min with 1 μM NE (B) or 1 μM clonidine (D), without or after 1-h preincubation with LY-294002 (LY, 10 μM) or yohimbine (Yoh, 0.1 μM). Phosphorylated PIP3 was quantified by radiography and densitometry, and PI3Kγ activation in treated tissues was expressed relative to non-treated tissue controls (bar graph, n = 3). *, *P *< 0.05 *versus *controls, #, P < 0.05 *versus *veins treated with NE or clonidine.

To determine whether NE directly activates PI3Ks, we measured phosphorylation of Akt in control vein segments and in tissues incubated with exogenous NE (1 μM, 3 min). Incubation with NE increased phosphorylation of Akt about 2-fold the basal (Fig. 7A). When smooth muscle strips were preincubated with wortmannin (100 nM, 1 h) or LY-294002 (10 μM, 1 h), NE failed to increase phosphorylation of Akt. These results indicate that NE-elicited phosphorylation of Akt in mesenteric vein requires activation of PI3Ks.

As shown in Fig. 1 and Fig 3, incubation of veins with yohimbine significantly reduced both the NE-induced and clonidine-induced contractions as well as the SMD elicited by EFS. These observations raise the possibility that the EFS and exogenous NE-induced activation of PI3Ks may be mediated by α_2_-adrenoceptors. To test this possibility, we assayed phosphorylation of Akt (Fig. 7C) in tissues incubated with clonidine (1 μM, 3 min) in the absence or presence of yohimbine (0.1 μM, 1 h). Clonidine alone increased phosphorylation of Akt to 2.3-fold the basal phosphorylation and this increase was abolished by inhibition of α_2_-adrenoceptors with yohimbine and by inhibition of PI3Ks with LY-294002. These data indicate that α_2_-adrenoceptors are coupled to PI3Ks and their downstream target Akt.

Previous experiments have shown that PI3Kγ mediates signaling downstream of GPCRs [18,33]. To test whether NE activates PI3Kγ, we incubated mesenteric vein with exogenous NE, then immunoprecipitated PI3Kγ and used the immunoprecipitated kinase to phosphorylate phosphatidylinositol (PI) *in vitro*. As shown in Fig. 7B, stimulation with NE increased the amount of PI3P (reaction product), suggesting that NE activated PI3Kγ. NE-mediated phosphorylation of PI was reversed in strips preincubated with LY-294002, indicating that it was a PI3K-dependent event. To test whether α_2_-adrenoceptors are linked to activation of PI3Kγ, we analyzed the activation of PI3Kγ in veins incubated with clonidine (1 μM) in the absence or presence of yohimbine (0.1 μM). As shown in Fig. 7D, clonidine activated PI3Kγ to 1.8-fold the basal, while yohimbine eliminated this activation. Thus, the activation of PI3Ks, and more specifically PI3Kγ, by exogenous NE and clonidine suggests that α_2_-adrenoceptors are coupled to PI3Kγ via activation of G-proteins.

### Stimulation of atypical PKC(s) contributes to the EFS-elicited SMD

**Figure 8:**
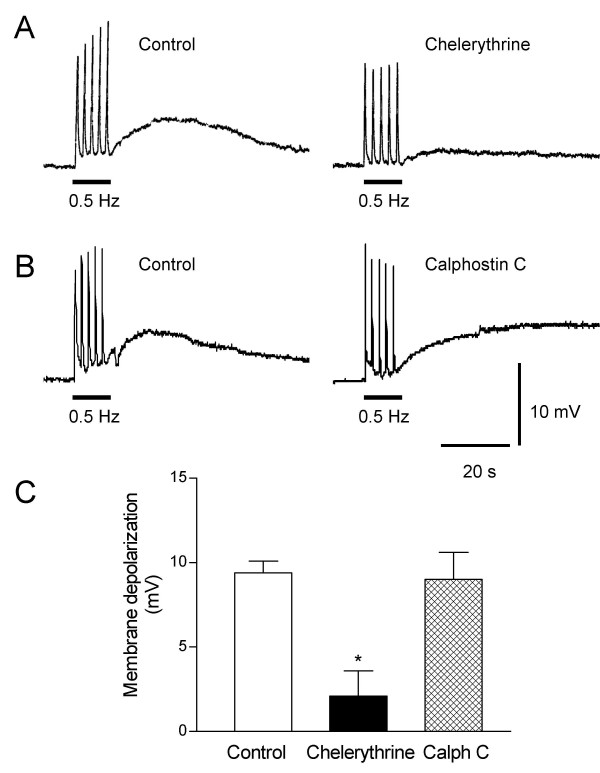
**The PKC inhibitor chelerythrine, but not calphostin C, reduces the EFS-evoked slow membrane depolarization in canine isolated mesenteric vein**. Intracellular recordings of membrane potential in response to EFS (0.1 ms, 13V, 10 s, 0.5 Hz) in control mesenteric veins (Control) or in tissues preincubated for 60 min with 1 μM chelerythrine (A) or 10 μM calphostin C (Calph C, B). The experimental data are presented as mean ± SEM, n = 3. *, *P *< 0.05 *versus *control. Scale bars apply to traces A and B.

PI3Kγ may be linked to membrane ion channels (i.e., ClC_Ca _and/or NSCC) via isozymes of the multifunctional PKC family. To test this possibility, we incubated mesenteric veins with the broad-spectrum PKC blocker chelerythrine (1 μM, 1 hour). Although chelerythrine reduced the response to 0.5 Hz EFS from 9.4 ± 0.7 mV to 2.1 ± 1.5 mV, the residual SMD remained unchanged in veins, incubated simultaneously with LY-294002 and chelerythrine prior to EFS (2.6 ± 1.3). These results not only implicate the existence of a PKC-dependent component in the EFS-induced SMD (Fig. 8A and 8C), but also suggest that PI3Kγ and PKC may signal to ion channels in a linear fashion. To narrow down the PKCs that function downstream of PI3Kγ, we used calphostin C, an inhibitor of classical and novel PKCs [32]. One-hour incubation of mesenteric veins with calphostin C (10 μM) failed to inhibit EFS-stimulated SMD (Fig. 8B and 8C). However, calphostin C significantly reduced the contractile responses to PMA (100 nM, 30 min incubation), which is mediated by classical and novel PKCs (not shown). Together, these results suggest that activation of atypical, rather than classical or novel PKC isozymes is involved in the SMD.

### Exogenous NE and clonidine activate PKCζ

**Figure 9:**
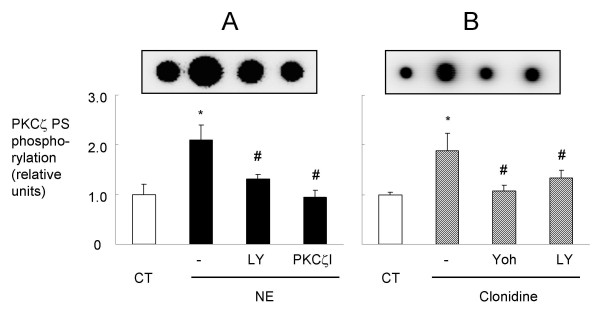
**NE and clonidine activate PKCζ in canine mesenteric vein**. Phosphorylation of a specific PKCζ peptide substrate (PKCζPS) *in vitro *by total protein extracts obtained from untreated controls (CT) and tissues incubates for 3 min with 1 μM NE (A) or 1 μM clonidine (B), without or after 60-min preincubation with LY-294002 (10 μM), myristoylated PKCζ peptide inhibitor (PKCζI, 50 μM) or yohimbine (Yoh, 0.1 μM). Phosphorylation of the PKCζ PS (i.e. density of the phospho-PS dots) was quantitated by radiography and densitometry (insets). Phosphorylation of PKCζSP in treated tissues is presented relative to non-treated tissue controls (bar graph, n = 3). *, *P *< 0.05 *versus *control; #, P < 0.05 *versus *veins treated with NE or clonidine.

Since exogenous NE and clonidine activated PI3Kγ, we tested whether these agents also activate PKCζ, a specific substrate of PI3Kγ [5]. Vein segments incubated with NE (1 μM, 3 min) and clonidine (1 μM, 3 min) caused activation of for a synthetic PKCζ peptide substrate *in vitro *to 2.11 and 1.89-fold the basal kinase activity of non-stimulated controls (Fig. 9A and 9B). The NE-induced activation of PKCζ was significantly reduced in tissues incubated with the PI3K inhibitor LY-294002 (10 μM) and was eliminated by a specific myristoylated peptide inhibitor of PKCζ (PKCζI, 50 μM) *in vitro *(Fig. 9A). Similarly, the clonidine-induced activation of PKCζ was eliminated in tissues preincubated with yohimbine and LY-294002 (Fig. 9B). These results indicate that stimulation of canine mesenteric veins with NE and clonidine is associated with activation of PI3Ks and a subsequent activation of PKCζ.

### Ion channel blockers and PI3K blockers do not inhibit release of NE

Reduction of the SMD could be the result of suppressed NE release from sympathetic nerve terminals in veins, incubated with protein kinase and ion channel blockers. To test this possibility, we assayed the EFS-evoked release of NE in superfusates collected during EFS in control veins and in tissues preincubated with each of the aforementioned blockers. The average EFS -evoked overflow of NE in tissue controls (16 Hz, 0.1 ms) was 122 ± 27 fmol/mg (n = 17). In preincubated tissues, the overflow of NE changed to (fmol/mg tissue) 236 ± 40 in the presence of NFA (n = 6), 480 ± 122 by NPPB (n = 6), 142 ± 2 by DIDS (n = 6), 144 ± 5 by wortmannin (n = 5), and 214 ± 38 by LY-294002 (n = 7). Therefore, none of these agents reduced the EFS-evoked overflow of NE, indicating that their effects on the EFS-induced SMD are not due to inhibited NE release but to activation of postjunctional (i.e., smooth muscle) mechanisms.

## Discussion

The NE-induced membrane depolarization is an essential requirement for opening VOCC, Ca^2+ ^entry and smooth muscle contraction, and hence it represents an important mechanism of autonomic neurovascular control. Previous studies [4] as well as the present work indicate that α_2_-adrenoceptors are involved in the SMD and the vasoconstriction of mesenteric vein. However, the downstream mechanisms that couple α_2_-adronoceptors to SMD remain undefined. For example, NE-induced activation of ClC_Ca _plays a key role in the associated vasoconstriction and presumed membrane depolarization in various vascular networks [11,16], but NSCC may also participate in the NE-induced vasoconstriction [17]. In the present study, we have expanded upon these previous works by directly measuring membrane potential in response to EFS of intact canine isolated mesenteric veins. We found that the SMD in response to EFS is frequency-dependent, and is sensitive to the fast Na^+ ^channel blocker TTX, to the inhibitor of neuronal N-type Ca^2+ ^channels ω-conotoxin GVIA [24], and to the selective antagonist of α_2_-adrenoceptors yohimbine. Consistent with previous works, therefore, our results indicate that EFS gives rise to a SMD, which is mediated by smooth muscle α_2_-adrenoceptors and hence is primarily mediated by NE, released upon action potential. Furthermore, the SMD of the vascular smooth muscle cell membrane appears to be mediated by channels sensitive to NFA, NPPB, and DIDS. Although these inhibitors target transporters with presumed high preference for Cl^- ^[8], they may also affect other ion channels, such as the inhibition of NSCC by DIDS [7]. Because incubation of mesenteric veins with the NSCC blocker Gd^3+ ^partially reduced the EFS-evoked SMD, our results suggest that activation of NSCC may indeed contribute to the EFS-elicited SMD in this blood vessel. None of the channel blockers reduced the release of NE thus demonstrating that the decrease of SMD is mediated by postjunctional smooth muscle mechanisms. The EFS-induced SMD remained unaffected by the InsP_3 _receptor inhibitors 2APB and xestospongin C, ruling out a substantial role of InsP_3 _receptor-mediated Ca^2+ ^release in this response. Therefore, smooth muscle cell chloride channels and/or NCSS appear to be the primary activities that mediate the SMD in the canine mesenteric vein.

Furthermore, we identified a novel signal transduction mechanism governing the slow membrane depolarization, which involves enzymes of the PKC family. First, we used two PKC inhibitors with distinct PKC isozymes selectivity. Chelerythrine, for example, acts on the conserved catalytic domain of PKC as a competitive inhibitor with respect to the phosphate acceptor and a non-competitive inhibitor with respect to ATP [32]. Therefore, chelerythrine can inhibit PKCs of all classes and effects mediated by them, including the slow depolarization in mesenteric veins. In contrast, calphostin C interacts with the regulatory domain of PKC by competing for the binding site of diacylglycerol and phorbol esters, but not Ca^2+ ^and phospholipids. Thus, calphostin C is more specific inhibitor for classical and novel, and less specific for atypical PKC [9,32]. In the present study, chelerythrine reduced, while calphostin C had no effect on the SMD. The lack of effect of calphostin C indicates that the EFS-induced membrane depolarization is most likely mediated by atypical PKC(s). We provided experimental support of this possibility by showing a distinct activation of the atypical PKCζ in mesenteric veins incubated with exogenous NE and clonidine. Although further studies are needed to identify the precise PKC isoform(s) contributing to the α_2_-adrenoceptor mediated SMD, the present study clearly suggests a PKC isozyme-specific regulation of the EFS- (and hence NE-) induced slow membrane depolarization in canine mesenteric vein.

The observation that atypical PKCs are involved in the SMC allowed us to speculate about the identity of some specific signaling molecules that function upstream of PKCζ, and could mediate the SMD as well. PI3Ks, and particularly PI3Kγ, was an obvious candidate for several reasons. Firstly, PI3Kγ is essential for activation of various ion transporters including L-type Ca^2+ ^channels [30] and metabotropic nonselective cation channels [31]. Secondly, PI3Kγ activates the phosphoinositide-dependent protein kinase 1 (PDK1), and although the latter can modulate classical (α and βI) and novel (δ and ε) PKC isoform, activation of atypical (ζ/λ/τ) PKCs, and particularly PKCζ, is a highly specific event downstream of PI3Kγ-PDK1 [28]. We experimentally supported the hypothesized role of PI3Ks by showing that the PI3K inhibitors wortmannin and LY-294002 prevent the NE and clonidine-dependent activation of PKCζ and the SMD. These data are consistent with a role of PI3K in the regulation of voltage-independent Cl^- ^channels, as well. PI3Kγ in particular is activated by G_βγ_ dimers, which are released upon dissociation of G_αβγ_ complexes following activation of GPCRs [18]. Our experimental data demonstrate activation of PI3Kγ in veins incubated with the G-protein coupled α_2_-adrenoceptor agonists NE and clonidine. Furthermore, since the NE and clonidine-mediated activation of PI3Kγ, PKCζ and of the SMD was prevented by yohimbine, our results demonstrate that activation of α_2_-adrenoceptors is required for activation of PI3Kγ and PKCζ, and possibly for activation of membrane Cl^- ^and/or NCSS channels and SMD in canine mesenteric vein. While the molecular identity of the membrane ion channels involved in these effects is presently elusive, our electrophysiological and biochemical data provide support to the possibility that activation of α_2_-adrenoceptors, PI3K and atypical PKC (possibly PKCζ) is essential for the regulation of the autonomic nervous system and vascular smooth muscle tone.

## Conclusion

In this study we provide functional and biochemical evidence that NE, released from postganglionic nerve terminals, activates postjunctional α_2_-adrenoceptors, PI3Ks and atypical PKCs in canine isolated mesenteric vein. Our results further suggest that specific isoforms of the PI3K and PKC families, i.e. PI3Kγ and PKCζ respectively, may participate in the signal transduction pathway that couples α_2_-adrenoceptors to membrane ion channels. This signal transduction pathway mediates slow membrane depolarization and vasoconstriction of canine mesenteric vein.

## Methods

### Tissue preparation

Seventy-four mongrel dogs of either sex (averaging 15 kg) were obtained from vendors licensed by the United States Department of Agriculture. The use of dogs was approved by the University of Nevada's Animal Care and Use Committee. The animals were sacrificed with an overdose of pentobarbitone sodium (100 mg/kg intravenously), as recommended by the Panel on Euthanasia of the American Veterinary Medical Association. Experiments were conducted with second and third order branches of the inferior mesenteric vein (0.7–1.2 mm in diameter), dissected and denuded of endothelium as outlined previously [21].

### Intracellular recording of membrane potential

Ring segments (7–10 mm long; 700–800 μM external diameter) were pinned out on the sylgard bottom of a 2 ml recording chamber perfused with Krebs solution (3 ml/min; 37°C; aerated with 95% O_2_/5% CO_2_) with the following composition (mM): 150 NaCl, 4.6 KCl, 1.2 MgCl_2_, 2.5 CaCl_2_, 24.8 NaHCO_3_, 1.2 KH_2_PO_4 _and 5.6 dextrose. Intracellular measurements were made through the adventitia of the vessel with fiber-containing borosilicate electrodes filled with 3 M KCl (70–100 MΩ resistance), as described previously [22]. EFS at supramaximal voltage with trains of square-wave pulses (0.1 ms pulse width) was applied at 0.1–2 Hz for 10 s by means of two parallel platinum electrodes on both sides of the vessel connected to a Grass S48 stimulator. Once well-defined and reproducible slow depolarizations were obtained, various drugs were applied to the superfusion solution according to the experimental protocol. The maximum depolarization was evaluated.

The effects of NFA (100 μM), NPPB (30 μM), DIDS (200 μM), 2 APB (50 μM), and xestospongin C (3 μM) were examined by recording membrane potential in response to EFS before and 30-min after the tissue was superfused with the drug. To assess the role of PI3K and PKC or the NSCC, tissues were superfused with wortmannin (100 nM), LY 294002 (10 μM), chelerythrine (1 μM), calphostin C (10 μM) or Gd^3+ ^(30 μM) for 60 min prior to the EFS. To produce appropriate time controls, EFS-mediated SMD were recorded periodically for 2–4 h in tissues not incubated with inhibitors. In some experiments tissues were perfused with NE (100 nM) for 30 s to induce SMD and changes in this response were monitored in the absence and presence of wortmannin (100 nM). In some experiments tissues were perfused with clonidine (1 μM) and changes in the membrane potential were monitored.

### Transmitter release experiments and HPLC assay of NE

Segments of endothelium-denuded mesenteric veins (52.5 ± 3.2 mg wet weight, n = 30) were placed in 200-μl BRANDEL superfusion chambers as previously described [23]. After 45 min equilibration, the tissues were subjected to a 30-seconds "conditioning" stimulation with a train of square wave pulses of 0.3 ms duration and a frequency of 4 Hz. Thirty minutes later the blood vessels were subjected to EFS for 60 s with a train of suprathreshold pulses of 0.1 ms duration at 16 Hz. Samples of the superfusion solution were collected *before *the electrical stimulation (resting overflow) and *during *the electrical stimulation (electrically evoked overflow) in ice-cold test tubes. Samples were analyzed for NE content by high performance liquid chromatography (HPLC) technique in conjunction with electrochemical detection [23].

After the equilibration period the tissues were superfused either with wortmannin (100 nM), or LY-294002 (10 μM), or chelerythrine (1 μM), or calphostin C (10 μM) for 60 min prior to EFS. In some experiments tissues were superfused with NFA (100 μM), NPPB (30 μM), or DIDS (200 μM) for 30 min prior to EFS. Only one drug was tested in each tissue.

### Mechanical responses

Ring preparations (3 mm long) were mounted in 3 ml organ baths by inserting two stainless steel triangle mounts into the lumen, and force displacements were further investigated as described previously [21]. The baths contained Krebs solution, which routinely contained indomethacin (1 μM) and Nω-nitro-L-arginine (l-NNA, 100 μM) to block potential residual effects of the endothelium and to eliminate possible time dependent effects due to activation of inducible nitric oxide synthase (iNOS) and/or cyclooxygenase. Thus, the contractile responses to KCl and nerve stimulation were reproducible over many hours when these blockers were included in the bathing solution. A resting force of 0.5 g was applied to the vein segments. This was found to stretch vessels to near the optimum length for tension development. After equilibrating the tissues [21], concentration-response relationships were obtained by cumulative addition of increasing concentrations of the α_1_-adrenoceptor agonist methoxamine in the absence and presence of 2APB (50 μM, 30 min pretreatment). Contractile responses elicited by methoxamine, NE and clonidine were expressed as a percentage of the contractile response produced with 70 mM KCl. The 70 mM KCl solution had the following composition (mM): 84.6 NaCl, 70 KCl, 1.2 MgCl_2_, 2.5 CaCl_2_, 24.8 NaHCO_3_, 1.2 KH_2_PO_4 _and 5.6 dextrose. Control experiments were carried out to determine the consistency of the contractile response to repeated applications of KCl (70 mM) over the duration of an average treatment protocol. The relative potency of methoxamine was determined by the concentration producing half-maximal effect. In some experiments, the effect of KCl (70 mM) was tested in the presence of nicardipine (1 μM). In other experiments, tissues were contracted with phorbol 12-myristate 13-acetate (PMA, 100 nM) in the absence or presence of chelerythrine (1 μM) or calphostin C (10 μM). In some experiments contractile responses to either exogenous NE or clonidine (both 0.05–10 μM) were monitored in the absence or presence of yohimbine (0.1 μM for 30 min).

### Preparation of tissue homogenates and cytosolic fractions

Total protein extracts were prepared by glass-glass homogenization of mesenteric veins, pulverized under liquid nitrogen, with a buffer composed of (mM): 10 Tris-HCl (pH 7.4), 5 EDTA, 5 EGTA, 10 sodium pyrophosphate, 10 NaF, 1 sodium orthovanadate, 0.1 AEBSF and 0.001 leupeptin. Insoluble material was pelleted by centrifugation at 3,000 g (S3 supernatants) for 5 min at 4°C. S3 supernatants were transferred into clean tubes and centrifuged at 120,000 g for 60 min at 4°C to obtain S120 supernatants, which were used for assay of PI3K and PKC activity.

### PI3K activation assay

Activation of PI3K was assayed by the phosphorylation of the downstream protein kinase Akt. Equal amounts of total supernatant protein (30 μg) were resolved by SDS-PAGE, transferred onto nitrocellulose membranes, and total and phospho-Ser473-Akt were assayed by immunoblotting, using a rabbit polyclonal or mouse monoclonal antibodies, respectively. Blots were scanned to obtain images and the immunoreactive bands were analyzed by densitometry, using the Quantity One software (BioRad). Changes in protein phosphorylation were calculated by normalizing the band density phospho-Akt to total Akt, and then were presented relative to the untreated group controls.

### PI3Kγ activation assay

PI3Kγ was immunoprecipitated from S120 supernatants with a mouse monoclonal antibody, immobilized on Protein A/G agarose plus beads (Santa Cruz Biotechnology, Inc.). Kinase activity was assayed by phosphorylation of phosphatidylinositol (PI) *in vitro*, as described previously [34].

### PKCζ activation assay

PKCζ activation was assayed by *in vitro *phosphorylation of a synthetic peptide substrate (PKCζPS), ERMRPRKRQGSVRRRV [15]. S120 fractions from control and treated tissues were used as enzyme sources. The phosphorylation reactions were stopped by cooling on ice, and 10 μl reactions were spotted on P81 filter strips. Excess radioactivity was removed from the filters by 3 washes with 75 mM *ortho*-phosphoric acid and a final wash with ethanol. The filters were air-dried prior to radiography and spots were quantified by densitometry, using a BioRad Model 525 Molecular Imager. In order to verify PKCζ-specific phosphorylation of the substrate, in some phosphorylation reactions we added a specific myristoylated PKCζ peptide inhibitor (PKCζI, 50 μM, Calbiochem).

### Drugs

NFA, NPPB, wortmannin, LY 294002, DIDS, nicardipine, norepinephrine hydrochloride, tetrodotoxin, phentolamine, xestospongin C, gadolinium chloride, PMA (Sigma-Aldrich), 2 APB (Tocris), PKCζPS and PKCζI (Calbiochem), chelerythrine, calphostin C (Biomol), general and phospho-specific anti-Akt antibodies (Cell Signaling Technology, Inc.), phosphatidylinositol (PI, Avanti). Gadolinium solutions were prepared fresh each day, essentially as described elsewhere [10]. Wortmannin, LY-294002, chelerythrine, calphostin C, PMA were dissolved in dimethylsulfoxide (DMSO) and diluted in double distilled water or Krebs solution. The final concentration of DMSO was less than 0.1 % DMSO.

### Statistical analysis

Data are presented as means ± SEM. Means were compared by analysis of variance (one-way ANOVA) (GraphPadPrism v. 3, GraphPad Software, Inc.). A probability value of less than 0.05 was considered significant. In the intracellular recording and mechanical response experiments, n refers to the number of rings, and hence dogs, included in each experimental group.

## Authors' contributions

Author IAY participated in the PI3K activation, PI3Kγ activation, and PKCζ activation assays. Author VM-Y participated in the membrane potential, NE release and mechanical activity experiments and designed and coordinated the study. All authors participated in drafting the manuscript.

All authors read and approved the final manuscript.
